# Empirically Informed, Idiographic Networks of Concordant and Discordant Motives: An Experience Sampling Study With Network Analysis in Non-Clinical Participants

**DOI:** 10.32872/cpe.12305

**Published:** 2025-05-28

**Authors:** Thies Lüdtke, Fabian Steiner, Thomas Berger, Stefan Westermann

**Affiliations:** 1 Department of Human Medicine, MSH Medical School Hamburg, Hamburg, Germany; 2 Institute of Sustainability Psychology, Leuphana University Lüneburg, Lüneburg, Germany; 3 Department of Clinical Psychology and Psychotherapy, Institute of Psychology, University of Bern, Bern, Switzerland; 4 Department of Psychology and Psychotherapy, Witten/Herdecke University, Witten, Germany; Philipps-University of Marburg, Marburg, Germany

**Keywords:** consistency theory, approach, avoidance, motive, concordance, conflict

## Abstract

**Background:**

Case formulations and treatment planning mostly rely on self-reports, observations, and third-party reports. We propose that these data sources can be complemented by idiographic networks of motive interactions, which are empirically derived from everyday life using the Experience Sampling Method (ESM). In these networks, positive edges represent concordance of motives whereas negative edges indicate discordance. Based on consistency theory, which states that discordance emerges when the activity of one motive (e.g., ‘affiliation’) is incompatible with the activity of another motive (e.g., ‘autonomy’), we hypothesized that discordance would be associated with subclinical depressive symptoms.

**Method:**

Fifty-one undergraduates completed a six-day ESM assessment period with 6 assessments of motive satisfaction per day. Based on the ESM data, idiographic networks of the seven most important motives per person were computed using mlVAR (https://doi.org/10.32614/CRAN.package.mlVAR). We extracted indices of motive dynamics from each person’s network, namely the strength of negative edges compared to the overall network strength as well as the values of the single most negative and positive edges. These indices were then used to predict subclinical depressive symptoms, controlling for overall motive satisfaction.

**Results:**

Discordant, conflicting motive relationships made up only 6% of network strengths, indicating high concordance overall. Neither conflict index predicted subclinical depressive symptoms but maximum concordance was associated with lower subclinical depressive symptoms. Motive satisfaction was a significant predictor across models.

**Conclusion:**

The applicability and clinical utility of the motive network approach was promising. Insufficient variance due to a healthy sample and the small number of observations limit the interpretability of findings.

## Highlights

- A novel approach to estimating idiographic networks of motives is introduced.
- Network links between motives indicate discordance (= conflict) or concordance (= compatibility).
- Motive satisfaction and concordance were associated with lower subclinical depressive symptoms.
- Unexpectedly, motive conflicts were not associated with subclinical depressive symptoms.

## Theoretical Background

For decades, therapists have relied on the same data sources for case conceptualization and treatment planning (Eells, 2007), namely questionnaires or clinical interviews, observations (e.g., nonverbal behavior), and information from third parties, such as family members. These data are undoubtedly indispensable but their ‘ecological validity’, meaning the applicability to real-world situations (Kihlstrom, 2021), can be questioned. One way to supplement traditional methods of case conceptualization is the structured assessment of patients’ experiences and behaviors in daily life, a method referred to as the Experience Sampling Method (Larson & Csikszentmihalyi, 2014), which relies on repeated self-report assessments, for example using smartphones. The feasibility of person-specific ESM studies has been demonstrated (Bak et al., 2016; Thonon et al., 2020; Wichers et al., 2016) and the necessary assessment tools (i.e., smartphones) are widely available (VuMa, 2023). Nonetheless, therapists seldom make use of this method beyond paper-pencil diaries in clinical practice, likely due to heavy caseload and lack of financial incentives.

Experience Sampling results in datasets which allow the estimation of both group-based and idiographic (Molenaar, 2004) models. One statistical approach, which is applicable to both types of models, is the so-called network approach (Borgatti et al., 2009), which has gained popularity within psychological research over the past years (Fried et al., 2017; Robinaugh et al., 2020). In network models, constructs are represented as *nodes* and *edges*. Nodes are the variables of interest, often symptoms (Borsboom, 2017), whereas edges represent statistical relationships between them, computed, for example, as partial correlations. Such networks can take many forms, such as social networks in which nodes represent people (Tabassum et al., 2018) or semantic networks in which nodes represent semantic or lexical units (Christensen & Kenett, 2023). In the present study, nodes represent psychological constructs whereas edges represent the partial correlations between these constructs. One of their major advantages is that networks can be graphically visualized, making them accessible to health professionals and patients alike (Bak et al., 2016). As Bak et al. (2016) phrase it, “discussing ESM results with a patient offers clues as to why and when symptoms vary, given certain stressors and contexts, with clues for protective mechanisms or coping strategies” (p. 8).

Why are ESM-based, idiographic assessments of patients’ experiences and behaviors in daily life not already a standard tool for therapists? We argue that – in addition to above-mentioned reasons – the information that is usually assessed in idiographic networks is not useful enough in aiding the conceptualization and individualization of psychotherapy. Although symptom-focused networks help to understand the centrality of certain symptoms and strength of symptom associations (e.g., Beard et al., 2016), they provide limited information about the underlying processes that may have led to their formation or maintenance. Given that psychological interventions are often targeted at mechanisms rather than symptoms (e.g., metacognitive biases; Moritz & Woodward, 2007), symptom networks may have limited clinical utility (current network approaches have other shortcomings, too, such as limited utility in ordinal data, see Borsboom et al., 2021). In line with that, Scholten, Lischetzke, and Glombiewski (2022) recently proposed a combination of functional analytic and experience sampling approaches. Here, we use network theory to assume a motive-focused view that answers questions such as *Does the patient value independence or confirmation by others*, *Does the patient seek intimacy and does she/he receive it*, and most importantly *Do the patient’s motivational goals clash*? Against this background (Westermann et al., 2019) we propose that a more instructive source of information for therapists is the interplay of patients’ motives in their daily life, particularly when these are in conflict.

The term ‘motive’ can be conceptualized as an active process that directs attention, cognition, and action (e.g., Schultheiss & Brunstein, 2005). According to Grawe (2004), motives serve as means to satisfy basic needs, such as the need for relatedness or autonomy (Deci & Ryan, 2000), and to protect them from violation. One can distinguish between approach and avoidance motives. Approach motives aim at creating or maintaining appetitive, need-satisfying experiences, such as being liked by others (affiliation). In contrast, avoidance motives are directed towards preventing or ending aversive, need-violating experiences, such as being criticized (self-esteem), that a person wants to avoid (Coats et al., 1996; Grosse Holtforth, 2008). According to consistency theory, motive satisfaction (i.e., ‘congruence’; Grawe, 2004) describes a state in which an approach motive is fulfilled (e.g., experiencing oneself as belonging) or the aversive experience is averted (e.g., avoiding being criticized), whereas motivational incongruence is characterized by the dissatisfaction of one’s motive, the latter being associated with mental health issues, such as loneliness (Gable, 2006) or the risk of chronicity of anxiety disorders (Struijs et al., 2018).

When the satisfaction of one motive supports satisfaction of another motive, they are in a concordant relationship with each other. In contrast, a discordant relationship emerges when the satisfaction of one motive reduces the satisfaction of another motive. For example, belonging and being autonomous can be mutually exclusive, as assertive behavior can be accompanied by discontent of others whereas overly cooperative behavior can violate one’s self-determination. Such motivational conflicts due to competing motives give rise to what is called ‘discordance’ within the framework of consistency theory. Discordance, in turn, is hypothesized to function as an ‘internal’ stressor (similar to cognitive dissonance; Festinger, 1957) that can facilitate the formation and maintenance of psychopathology (Grawe, 2004). In line with this, psychotherapy is accompanied by a reduction of incongruence (e.g., Berking et al., 2003) and psychological conflicts of goals (more broadly defined and not restricted to motives) are inversely associated with psychological well-being (Gray et al., 2017).

We propose that ESM-based measurements of motive satisfaction and conflict are ideally suited to augment established methods of treatment planning because they allow assessing motivational dynamics *in vivo* and in personally relevant situations, unaffected by retrospective recall biases (Ben-Zeev & Young, 2010; Urban et al., 2018). According to our approach, the interplay of motives in daily life can be represented as an idiographic network, in which nodes depict motives and edges constitute concordant (i.e., positive) versus discordant (i.e., negative) relationships between motives, potentially providing information that helps to personalize treatment and improve the therapeutic relationship (Caspar, 1997). Importantly, we propose that motivational conflicts can be empirically assessed even when participants are not able to report those associations in self-reports (e.g., due to a simple lack of explicit representation or due to defense mechanisms in a psychodynamic sense, see Blanco et al., 2023). Table 1 provides an overview of relevant motive-related terms and how they were operationalized in the network methodology.

**Table 1:** Motive-Related Terms and Their Network Operationalization

Term	Network operationalization
Motive	Node
(In)congruence	Momentary value of node (0: incongruence, 9: congruence)
Motive interaction	Edge
Concordant motives (mutualistic interaction of two motives over time)	Positive edge
Discordant/conflicting motives (competitive interaction of two motives over time)	Negative edge
Concordance / Discordance	Summary statistics of edge weights in the network (e.g., negative edge weights divided by all edge weights; see methods section)

The clinical utility of motive dynamics is not limited to a certain diagnosis or symptom spectrum (Grosse Holtforth & Grawe, 2004). However, for the first-time validation of the proposed motive network approach, we focus on depressive symptoms. According to the reinforcement theory of depression (Ferster, 1973; Lewinsohn & Graf, 1973; Lewinsohn & Libet, 1972), a low rate of response-contingent positive reinforcement through rewarding activities is a key factor in the development and maintenance of depressive symptoms (Lewinsohn, 1974, p. 151). Within our motive network approach, activities are perceived as rewarding if they satisfy an individual’s motives (Brockmeyer et al., 2015), but if the satisfaction of one motive is accompanied by the dissatisfaction of another motive (i.e., motivational discordance), activities may lose their reinforcing properties. Enduring states of conflict due to discordant motives could lead to diminished reinforcement and thus depressive symptoms whereas motivational concordance may be a protective factor.

In sum, we hypothesized that individuals with higher subclinical depressive symptoms would display stronger motive conflicts, operationalized as the proportion of negative edges, as well as the magnitude of the largest negative edge within the individual’s network. Relatedly, we hypothesized that concordance, operationalized as the magnitude of the strongest positive edge would be associated with *reduced* subclinical depressive symptoms. We excluded avoidance motives from the analyses because they are empirically associated with psychopathology (Grosse Holtforth, 2008), which may confound analyses.

## Method

### Recruitment

We recruited undergraduate psychology students who were compensated with course credits. Participants were eligible for participation if they were at least 18 years of age and did not report any mental disorder. The ethics committee of the Faculty of Human Sciences at the University of Bern approved the study (#2016-05-00006).

The present study was part of a larger project (Lüdtke & Westermann, 2023). Sample size considerations were based on recommendations for multilevel modelling (Maas & Hox, 2005) given the nested data structure of repeated measurements within participants. The target sample size was set to *n* = 50 level two cases (i.e., participants) to avoid biased estimates of second-level standard errors (Maas & Hox, 2005). No stopping rule for data collection was applied. Given that our initial power analysis focused on maximizing level two cases, the number of data points within participants (up to 36 assessments) was limited when compared to recommendations for within-person network estimation (Mansueto et al., 2023).

### Procedures

Assessments were conducted between August and November 2016. All participants provided written informed consent prior to participation. The baseline assessment consisted of an online survey as well as a face-to-face meeting. The online survey covered sociodemographic and clinical variables, approach- and avoidance motives, as well as subclinical depressive symptoms (see Baseline Measures section). During the face-to-face meeting, participants received a study smartphone and were instructed to carry it with them for six consecutive days. All functions of the smartphones were disabled except for the ESM survey application ‘movisensXS’, version 0.8.4211 (movisens GmbH, Germany).

### Measures

#### Baseline Measures

The German version of the Depression Anxiety Stress Scales (DASS-21; Nilges & Essau, 2015) was used to measure subclinical depressive symptoms. Participants rated how much each item applied to them over the past week (e.g., “I felt that life was meaningless”), with response options ranging from *not at all* to *very much, or most of the time* on a four-point scale. Internal consistencies ranged from α = .79 for the anxiety subscale to α = .87 for the depression subscale in the present sample. The DASS-21 can be used both in clinical and non-clinical samples (Antony et al., 1998).

The Inventory of Approach and Avoidance Motivation (IAAM; Grosse Holtforth & Grawe, 2000) measures the importance of motives from *not at all* to *extremely important*. The questionnaire was developed in a ‘bottom-up’ process based on patients’ case formulations (Grosse Holtforth, 2008) covering 14 approach- and nine avoidance motives on 94 items. For approach motives, internal consistencies range from .62 (self-reward) to .90 (affiliation) in psychology students (Grosse Holtforth & Grawe, 2000).

Whereas the IAAM measures motive importance, the incongruence questionnaire (INC; Grosse Holtforth & Grawe, 2003) measures the extent to which motives are implemented/satisfied in participants’ interactions with their environment (recoded to represent the degree of incongruence). Internal consistencies of INC subscales range from .71 to .89 in healthy participants (Grosse Holtforth & Grawe, 2003). For the present study, only approach motives were considered. The short version of the scale (INC-S; Grosse Holtforth & Grawe, 2003) consists of one item per motive and was used in the ESM assessment phase (see ESM Assessments section).

#### ESM Assessments

Participants completed six assessments of momentary motive satisfaction per day, for a period of six days, resulting in a maximum of 36 assessments per participant. Entries were defined as missing if they were dismissed, ignored, discontinued, or completed more than 30 minutes after the prompt. Each ESM assessment comprised 36 items. ESM assessments occurred at pseudo-random times between 9:30 a.m. and 9:30 p.m. with a minimum inter-assessment distance of 60 minutes. In addition, we offered the possibility to access the ESM assessment manually in case of a missed prompt. Each ESM assessment took approximately one minute to answer. Participants completed a total of 1,481 assessments, of which 1,406 assessments were prompted by the smartphone (95%), whereas 75 assessments were manually accessed by the participant within 30 minutes after a missed/ignored prompt (5%). Full adherence would have resulted in 36 x 51 = 1,836 datapoints, so 1,481 assessments correspond to 81% adherence. On average, participants responded to a prompt after 301 seconds (*SD* = 427 seconds, range = 1 to 1761 seconds, Median = 27 seconds). The distribution was skewed with half of the responses occurring within the first 30 seconds after the prompt. Most participants (*n* = 48; 94%) used the study smartphone for 6 days as intended. After completing the ESM phase, participants returned the study smartphones and completed a debriefing session.

Motive satisfaction was assessed six times per day using the short version of the Incongruence Questionnaire (INC-S; Grosse Holtforth & Grawe, 2003). We made slight adjustments to the INC-S. First, a ten-point Likert scale was administered to allow for a more fine-grained assessment of motive satisfaction while retaining the endpoints of the scale. Second, we adjusted the wording to capture momentary motive satisfaction rather than general motive satisfaction: “*Below you will find a list of different pleasant and unpleasant experiences. Please indicate how sufficiently you have been able to realize the more pleasant ones since the last survey (part 1) and how much the more unpleasant ones apply to you since the last survey (part 2)*” (translated from German). The internal consistency of approach motive satisfaction has been reported as good (.84; Grosse Holtforth & Grawe, 2003) and the correlation between the short and the long version of the INC was high in the present sample (*r* = .62, *p* < .001), illustrating the validity of the INC-S in the ESM setting (for both scales, the seven most important motives based on the IAAM were used).

The INC-S was accompanied by a short version of the Positive and Negative Affect Scales (PANAS; Watson et al., 1988), as well as items on the situational context (see Lüdtke & Westermann, 2023). We used only one of the PANAS items in exploratory analyses to examine the effect of momentary motive satisfaction on concurrent sadness, namely “In the present moment, I feel…” with the response options ranging from *unhappy* to *happy* on a seven-point scale.

### Analyses

First, we computed idiographic networks of motive satisfaction separately for each participant. To do so, we identified the seven most important motives that participants endorsed in the IAAM and entered the corresponding INC-S items into a network. Selecting the most important motives per participant ensured that potential motive conflicts were personally relevant and it helped to reduce model complexity (i.e., fewer nodes), which is recommended for low numbers of observations (Mansueto et al., 2023). Conceptually, the resulting networks represented concordant or discordant interactions (i.e., edges) between the satisfaction of idiosyncratically relevant motives (i.e., nodes) within a participant across the ESM period. Hence, a positive edge indicated that the satisfaction of one motive was accompanied by the concomitant satisfaction of another motive, whereas a negative edge indicated that whenever one motive was satisfied, another motive was less satisfied. Network estimation was conducted using the mlVAR package in R, version 0.5.^1^1CRAN link: http://cran.r-project.org/package=mlVAR Github link (developmental): http://www.github.com/SachaEpskamp/mlVAR We computed contemporaneous networks, which indicate how the satisfaction of one motive is associated with the satisfaction of another goal at the same time (i.e., partial correlations). The mlVAR package estimates these networks by extracting the residuals of time-lagged temporal models based on non-correlated random effects. Model estimation relied on linear mixed effects (lmer), the contemporaneous network estimation was set to “orthogonal”, without prior standardization. Node-specific fit indices provided are presented in the Supplementary Materials.

In the second step of the analysis, we extracted network parameters on motive concordance versus discordance from each model and examined their association with subclinical depressive symptoms on a group level (i.e., between persons). First, we calculated the proportion of negative edges relative to total edge strength in the network, henceforth referred to as *conflict proportion*. To do so, we added up the absolute values of negative edge weights and divided them by the absolute values of all edge weights. The resulting index of discordance can be interpreted as the proportion of conflicting edge strength relative to the overall strength of edges within the network. Additionally, we extracted the strongest negative edge from each network, referred to as *maximum conflict*, as well as the strongest positive edge, referred to as the *maximum concordance*. The rationale for estimating the *maximum conflict* parameter was that one strong motivational conflict may have detrimental consequences because the conflicting motives cannot both be satisfied at the same time, resulting in over-active motives with ongoing activity and an overall lower level of congruence (e.g., Boudreaux & Ozer, 2013). For example, while autonomy and affiliation are in conflict, the self-esteem motive has fewer opportunities to become satisfied. The rationale for the *maximum concordance* parameter was that one strong concordant relationship between important motives allows for efficient behavior that results in high decreases of incongruence.

Network parameters of motive discordance and concordance were then used to predict subclinical depressive symptoms (DASS-D; Nilges & Essau, 2015) in OLS regression models. Motive satisfaction across the ESM period (INC-S; Grosse Holtforth & Grawe, 2003) was added as a covariate to all models. A check of model assumptions revealed that the outcome was skewed due to a large proportion of participants reporting hardly any subclinical depressive symptoms, and a visual inspection of residuals indicated problems with heteroscedasticity and non-normality. As a log-transformation could not resolve the issue, we resorted to bias-corrected and accelerated (BCa) bootstrap confidence intervals and corresponding *p*-values (based on 5000 samples), which are robust to violations of assumptions, such as non-normality (Field, 2009, p. 163). All tests were two-sided with conventional *p*-values of .05.

Following confirmatory analyses, we conducted an exploratory linear mixed model (LMM) analysis to examine the moment-to-moment effects of motive satisfaction on concurrent mood. LMM allowed us to examine how the level of motive satisfaction, that is (in)congruence, relates to momentary affective states measured concomitantly. Thus, the LMM analyses answered the question whether – within participants – a moment in which motives were more strongly satisfied was associated with better mood as compared to a moment in which motives were less satisfied (and vice versa). LMM analyses account for the clustering of time points nested within individuals (Twisk, 2019, p. 150). The model included a random intercept, and it relied on maximum likelihood estimation. Averaged as well as momentary person-mean-centered motive satisfaction were entered as predictors (a so-called hybrid model; Twisk, 2019, p. 139).

## Results

### Participant Characteristics and Adherence

A priori it was determined that participants with fewer than 12 completed assessments (i.e., 33%) would be excluded from analyses. Out of *n* = 55 participants, four (7%) completed less than 12 timely ESM assessments, leaving *n* = 51 participants. Baseline characteristics are presented in Table 2.

**Table 2:** Baseline Characteristics (n = 51)

Baseline Characteristic	
Age; *M* (*SD*)	21.8 (2.1)
Gender; female/male	45/6
Years of education; *M* (*SD*)	14.3 (1.8)
DASS-21-D; *M* (*SD*)	1.6 (0.6)

*Note*. DASS-21-D = DASS-21 depression subscale.

### Importance, Satisfaction and Conflict of Motives

**Figure 1:**
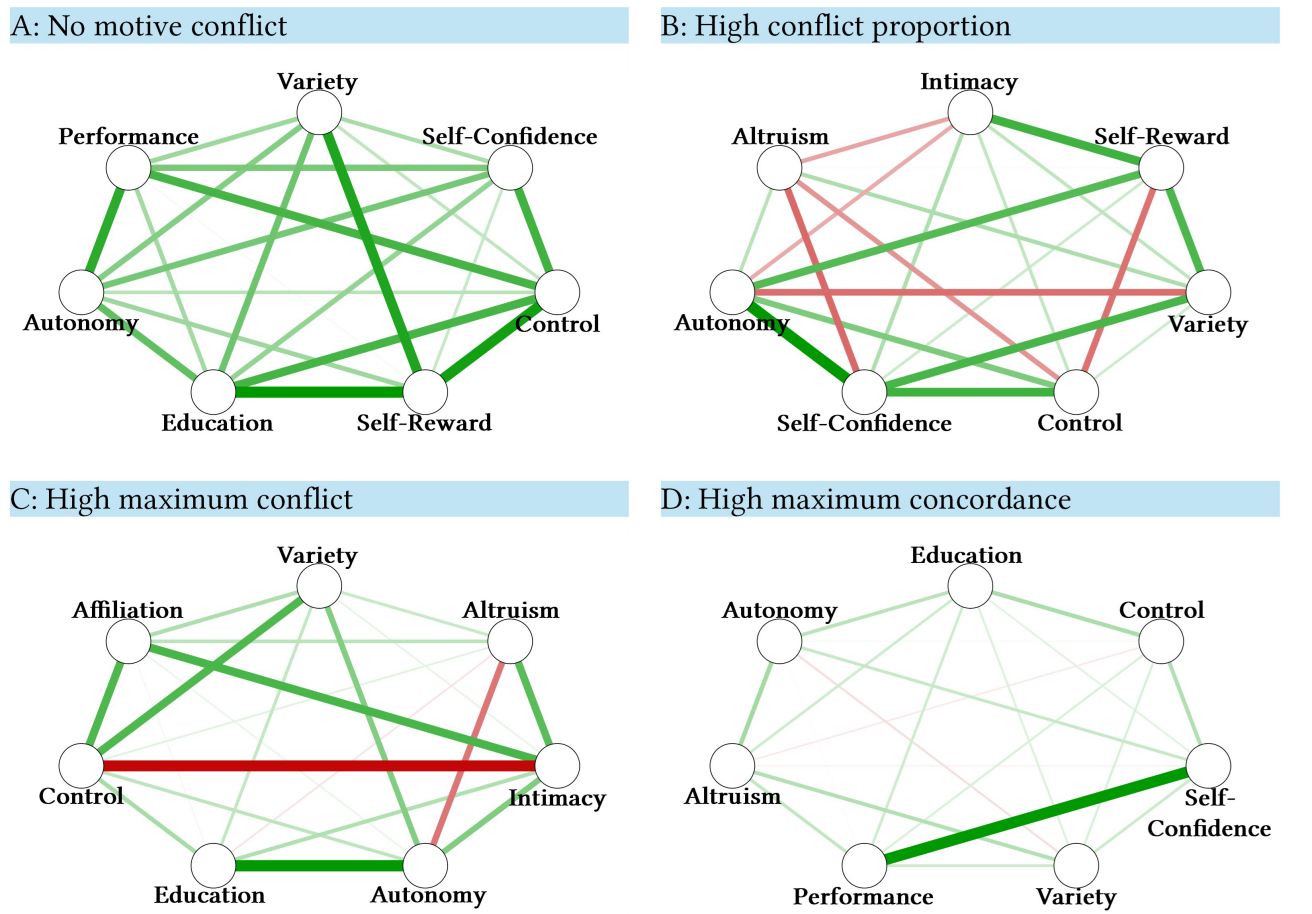
Four Exemplary Idiographic Motive Networks *Notes.* Network graphs of four participants are depicted for illustrative purposes. Green lines (i.e., edges) represent concordant relationships between motives whereas red lines represent discordant relationships. The thickness of edges is determined by the strength of the association. Motive network A (Participant 72) depicts uniformly positive edges, indicating no conflict, motive network B (Participant 71) depicts multiple moderately negative edges (29% conflict proportion), motive network C (Participant 41) shows few but strong negative edges (-.33 maximum conflict), and motive network D shows a case of maximum concordance (.61 maximum concordance).

The motives that were most often represented in the idiographic networks were ‘confidence’ and ‘variety’ (both part of 43 idiographic networks; 78%), followed by ‘intimacy’ (76%), ‘autonomy’ (65%), and ‘self-reward’ (64%). The ‘status’ goal was the least prominent, as it was represented only in one idiographic network (2%). The mean-ratings in the IAAM support the importance of the aforementioned motives, with ‘self-confidence’ receiving the highest average rating (*M* = 4.28 on the 5-point scale, *SD* = 0.51), followed by ‘autonomy’ (*M* = 4.21, *SD* = 0.53) and ‘intimacy’ (*M* = 4.20, *SD* = 0.65), whereas ‘status’ received the lowest rating (*M* = 2.47, *SD* = 0.56). Across the ESM period, the motive with the highest grand-mean satisfaction was autonomy (*M* = 7.18 on the 10-point scale, *SD* = 1.95), followed by control (*M* = 7.03, *SD* = 1.87). However, a variance component analysis (‘null model’) revealed that participants differed significantly in terms of motive satisfaction across the ESM-period (Wald Z tests: *p*’s < .001). Figure 1 depicts four exemplary idiographic motive networks.

Across the sample, motives were mostly concordant, with an average conflict proportion of 6.2% (*SD* = 6.7%; range = 0 to 29%). Of note, *n* = 10 participants showed no conflict at all, and the distribution was skewed due to low values. For maximum conflict, the resulting distribution was skewed towards zero as well, with a mean of *r* = -.09 (*SD* = .09; range: 0 to -.33). Exemplary time series of motivational conflict versus cooperation are presented in the Supplementary Materials.

### Associations Between Motive Concordance as Well as Discordance and Subclinical Depressive Symptoms

We conducted three linear regression analyses (see Table 3). The first model examined whether the conflict proportion predicted subclinical depressive symptom severity, whereas the remaining two models examined the effect of the maximum conflict and maximum concordance, respectively. In all models, motive satisfaction was entered as a covariate.

**Table 3:** Network-Derived Motive Satisfaction, Concordance and Discordance as Predictors of Subclinical Depressive Symptoms

Predictor	*b*	*SE*	*t* (*df*)	*p*	Bootstrap 95%-CI; *p*
Motive satisfaction	-0.139	0.059	2.358 (48)	.023	[-0.258, -0.013]; *p* = .047
Proportion of negative edges	0.374	1.269	0.295 (48)	.770	[-2.341, 3.014]; *p* = .792
Motive satisfaction	-0.167	0.057	2.921 (48)	.005	[-0.286, -0.052]; *p* = .012
Most negative edge	0.995	0.940	1.058 (48)	.295	[-0.517, 2.405]; *p* = .198
Motive satisfaction	-0.165	0.054	3.072 (48)	.003	[-0.285, -0.051]; *p* = .008
Most positive edge	-1.136	0.643	1.765 (48)	.084	[-2.119, -0.038]; *p* = .021

*Note*. Intercept coefficients not displayed; due to heteroskedasticity and non-normality of residuals, Bootstrap confidence intervals and *p*-values are provided.

Contrary to our hypotheses, neither conflict proportion nor maximum conflict predicted subclinical depressive symptoms (see Table 3). In contrast, the maximum concordance predicted subclinical depressive symptoms in that higher maximum concordance was associated with reduced subclinical depressive symptoms in models using robust bootstrapping. Motive satisfaction, which was added as a covariate, was a significant predictor of subclinical depressive symptoms across models in that more satisfaction was associated with less subclinical depressive symptoms.

### Exploratory Analyses

It was surprising that the maximum concordance was associated with subclinical depressive symptoms whereas the maximum conflict was not. Our conjecture was that the null effects regarding motivational conflict might be related to a lack of discordant motivational relations (and thus variance). When we excluded participants from the analyses who displayed not one subclinical depressive symptom or motive-conflict (*n* = 15), maximum conflict was associated with subclinical depressive symptoms according to bootstrapping (*b* = 1.744, 95% Bootstrap-CI [0.054, 3.329]), but not conventional significance testing (*p* = .131). Tentatively, these findings suggest that a lack of variance may have contributed to null results at least partly and that it may be worthwhile to examine effect of maximum conflict in participants who display more motive conflicts.

Confirmatory analyses indicated that participants with higher average motive satisfaction across the six-day ESM period reported lower subclinical depressive symptoms. Possibly, this effect emerged because motive satisfaction was immediately related to positive mood on a moment-to-moment level. Thus, we examined whether momentary within-person fluctuations of motive satisfaction were associated with contemporaneous affect (i.e., *unhappy* to *happy*) in a LMM analysis. The model controlled for the mean level of motive satisfaction (i.e., a ‘hybrid model’) to disentangle between- from within-person variance. Results suggested that, on a within-person level, an increase in motive satisfaction predicted more happiness (fixed effect: *b* = 0.406, *SE* = .024, *p* < .001), meaning that when a person felt that their motives were satisfied more than usually, they felt in fact happier.

## Discussion

The present study demonstrated the feasibility and, in part, the clinical utility of a novel network-based approach to quantifying motive concordance and discordance in participants’ everyday lives using Experience Sampling. Adherence (81%) was relatively high compared to other ESM studies (e.g., 76.8% in psychosis research; Deakin et al., 2022), and only four participants had to be excluded because of too few assessments. These data suggest that the proposed ESM-based motive network approach could be a feasible tool for therapists to learn about patients’ idiographic motivational conflicts in order to aid treatment planning. The clinical utility of the derived indices of concordance and discordance was supported partly, as higher maximum concordance (i.e., strong concordant relationships between at least two motives) and overall motive satisfaction were associated with reduced subclinical depressive symptoms whereas motive conflicts were not.

In terms of validity, the assessment method has proven to be promising because the correlation between trait incongruence assessed via self-report and the average incongruence in daily life was high but not redundant (*r* = .62). Visual representations of aggregated motive conflict (Figure 1) could serve as valuable sources of information for both therapists and patients. Inter-individual differences in idiographic network composition and everyday-life motive satisfaction support the usefulness of the idiographic approach.

Whereas network-derived concordance may be a protective factor, motivational conflicts showed no associations with subclinical depressive symptoms. It is possible that null effects were the result of the low number of observations within participants (i.e., 36 given full adherence) in combination with a low probability of occurrence of motivational discordance in the healthy student sample. A clinical sample would likely increase the likelihood of detecting more severe motive conflicts, which might be better suited to predict depressive symptoms. For example, edges and centrality strength show stronger correlations between internalizing symptom networks from the same population than between networks from clinical versus non-clinical samples (Funkhouser et al., 2020). Alternatively, when explaining the null finding one has to take into account that incongruence and discordance overlap empirically and theoretically, because discordance actually results in incongruence. Therefore, testing that discordance explains variance in depressive symptoms over and above incongruence is particularly conservative. Finally, an alternative psychological explanation for the null finding is, of course, that the motive conflicts that *are* related to depression are not captured with our network operationalization of motive conflict (e.g., due to motive incongruence not being consciously accessible due to avoidance motives and/or defense mechanisms).

### The Role of Motive Satisfaction

ESM-derived motive satisfaction emerged as a significant predictor of subclinical depressive symptoms across models. Interestingly, exploratory post hoc analyses suggest that momentary fluctuations in approach motive satisfaction were associated with momentary happiness. This result is in line with theoretical accounts of approach and avoidance motivation (Carver & Scheier, 2000), and it adds to findings that a lack of motive satisfaction is associated with reduced well-being, self-esteem, or joie de vivre (Grosse Holtforth & Grawe, 2003) between persons but also on a moment-to-moment level. In addition, this finding provides further evidence for the importance of resource activation in psychotherapy and a salutogenetic perspective on mental health in general (e.g., Munder et al., 2019). Consequently, the clinical utility of the assessment might lie in the identification of how an individual motive network can be driven to more concordance during psychotherapy.

### Limitations and Future Directions

First, the number of observations was low. Even 75 or 100 assessments are associated with low sensitivity (Mansueto et al., 2023), so 36 assessments in the present sample may have been too few to reliably estimate network parameters. However, we have made several design choices that were aimed at reducing the impact of small numbers of observations (Mansueto et al., 2023), namely limiting the number of nodes by selecting the seven most important motives per person, constraining analyses to contemporaneous networks, and using full information maximum likelihood estimation. One might argue that a more robust measure of motive interactions, such as simple correlations between motives, would be more appropriate. We decided against this option because, conceptually, the isolated relationship between two motives is not as informative as the interplay of all motives, which is illustrated by the fact that motivational (in)congruence is assessed using a combination of all approach and avoidance motives, respectively (Grosse Holtforth & Grawe, 2003). Unlike simple correlations, partial correlation networks represent associations between two nodes while controlling for all other information possible (Epskamp & Fried, 2018), which we consider a much more appropriate representation of overall concordance/discordance among motives.

Second, the student sample was educated, healthy, and mostly unaffected by motivational conflict according to our approach, resulting in skewed data and non-normally distributed errors. We addressed these issues with robust bootstrapping procedures but could not overcome the conceptual problem of floor effects. Third, the method of motive networks must be critically discussed. A motive network is based on an individual’s transaction with their environment. A conflict between motives might occur due to motive-related conflicts, but also due to challenges imposed by the environment, a skill deficit, or a combination of such factors. In the current study, it is not possible to partition those sources of variance, and it would be worthwhile to qualitatively assess how conflicts emerged and how they were experienced by participants in future studies.

Fourth, the theoretical framework we drew upon – consistency theory (Grawe, 2004) – is only one amongst many that are capable of informing motive conflicts. In the domain of psychodynamics, there are many theories that address motives and conflicts between motives. For example, the two-polarities model of personality development (Blatt, 2007) assumes two motivational processes: interpersonal relatedness and self-definition. In this model, psychopathology such as a depressive disorder can stem from a too strong focus on one of these processes accompanied by a neglect of the other. However, if one motivational process is active and the other is inactive all the time – in contrast to alternating activity –, there is no variance to be captured as motive conflict in experience sampling data. Thus, the theoretical perspective adopted here, and the resulting operationalization cannot capture the entirety of motive conflicts, limiting the interpretation of the findings. Lastly, the slightly adapted version of the INC-S which we used to assess congruence in daily life was not validated prior to the study.

There are several avenues for future research. First, the convergent validity of motive networks could be assessed using other data sources such as clinical interviews (e.g., OPD; Arbeitskreis zur Operationalisierung Psychodynamischer Diagnostik, 2023). Second, the test-retest reliability could be determined through repeated experience sampling phases (e.g., a month apart). Lastly, the qualitative evaluation of motive networks could be achieved by interviewing participants about their personal motive networks (e.g., individual concordant and conflicting pairs of motives and their face validity for the interviewees).

## Supplementary Materials

The supplement contains a table which lists all approach motives that were assessed in the present study including respective Intraclass Correlation Coefficients (ICCs), a table with fit indices for each node of the idiographic network models, as well as a figure with exemplary depictions of congruence of the autonomy and the affiliation motive over time for two participants (for access, see Lüdtke et al., 2025S).



LüdtkeT.
SteinerF.
BergerT.
WestermannS.
 (2025S). Supplementary materials to "Empirically informed, idiographic networks of concordant and discordant motives: An experience sampling study with network analysis in non-clinical participants"
[Additional information]. PsychOpen. 10.23668/psycharchives.16213
PMC1216369040519802

## Data Availability

The data that support the findings of this study as well as the code are available from the corresponding author, Stefan Westermann, upon reasonable request.

## Preregistration

The study was not pre-registered.

## Reporting Guidelines

The manuscript was prepared in accordance with the JARS-Quant guidelines for quantitative research. An overview of approach motives as well as fit indices of the network’s nodes are provided in the Supplementary Materials.
